# Effects of the Magnetic Resonance Imaging Contrast Agent Gd-DTPA on Plant Growth and Root Imaging in Rice

**DOI:** 10.1371/journal.pone.0100246

**Published:** 2014-06-19

**Authors:** Zan Liu, Junchao Qian, Binmei Liu, Qi Wang, Xiaoyu Ni, Yaling Dong, Kai Zhong, Yuejin Wu

**Affiliations:** 1 Key laboratory of ion beam bioengineering, Institute of Technical Biology and Agriculture Engineering, Hefei Institutes of Physical Science, Chinese Academy of Sciences, Hefei, P. R. China; 2 High Magnetic Field Laboratory, Hefei Institutes of Physical Science, Chinese Academy of Sciences, Hefei, P. R. China; Institute of Genetics and Developmental Biology, Chinese Academy of Sciences, China

## Abstract

Although paramagnetic contrast agents have a wide range of applications in medical studies involving magnetic resonance imaging (MRI), these agents are seldom used to enhance MRI images of plant root systems. To extend the application of MRI contrast agents to plant research and to develop related techniques to study root systems, we examined the applicability of the MRI contrast agent Gd-DTPA to the imaging of rice roots. Specifically, we examined the biological effects of various concentrations of Gd-DTPA on rice growth and MRI images. Analysis of electrical conductivity and plant height demonstrated that 5 mmol Gd-DTPA had little impact on rice in the short-term. The results of signal intensity and spin-lattice relaxation time (T1) analysis suggested that 5 mmol Gd-DTPA was the appropriate concentration for enhancing MRI signals. In addition, examination of the long-term effects of Gd-DTPA on plant height showed that levels of this compound up to 5 mmol had little impact on rice growth and (to some extent) increased the biomass of rice.

## Introduction

Root systems play an important role in plant nutrition and water absorption, as well as the synthesis and storage of metabolites. Examining the structure of the root system in a quantitative manner may lead to an understanding of its function. Compared to aboveground plant structures, roots are difficult to examine due to the complexity of the root growth environment and the limits of research methods and quantitative analysis. In addition,for many years, researchers have paid relatively little attention to the root system. Currently, efficient methods for root research are lacking. Obviously, better techniques are needed to study root systems.

Traditional techniques to study plant root are labor-intensive; simultaneously, many of these techniques are destructive and lack accuracy [1]. In recent years, some improved methods such as the minirhizotron technique, X-ray computed tomography (X-CT) and magnetic resonance imaging (MRI) have been applied to study the root system. Minirhizotrons provide root data in a non-destructive manner and can be used to immediately view and study fine roots [2]. However, the external tubes in minirhizotrons may cause a certain degree of soil disturbance, and the resulting root data may differ somewhat from standard data. The limitations of the resulting images make the root data incomplete and restricted. X-CT has been employed numerous times in root studies. Kaestner et al. [3] used X-CT to reconstruct the alder root network. Mooney et al. [4] discussed the basics, advantages and application of X-CT to plant research. However, X-rays are ionizing radiation and may therefore inhibit growth and cause damage and even necrosis in cells. Moreover, X-ray irradiation is incompatible with metabolite analysis [5]. Thus, X-CT can potentially damage the root system.

MRI has been widely used in medical research. The application of this technique to plant science research is still at the exploration stage. However, MRI has the advantage of being non-destructive and may potentially be used to detect physiological changes that occur in vivo [5]. Due to the differences in water content between plant tissues and the surrounding materials, MRI can be used to detect plant characteristics and to image different plant tissues. To date, many studies using MRI have been performed to study plant development [6], water dynamics in living plants [7], [8], and plant metabolism [9] and to functionally image the abiotic stress response [10], [11]. The big scene of root systems should be studied during all growth periods and high field magnetic resonance devices make such studies feasible.

To broaden the application of MRI to studies of rice roots, the MRI signal intensity of the root must be improved. In medical research, contrast agents are often used to improve the signal differences between normal and diseased tissue, as these agents increase the relaxation times of water protons [12]. However, few plant science studies have employed contrast agents. Zhong et al. [13] used the paramagnetic agents GdDTPA^2–^ and DyDTPA-BMA to examine maize root fragments, and they also observed NMR signals from intracellular and extracellular ^1^H_2_O. Eberhardt et al. [14] used GdCl_3_ as a contrast agent to image wood via magnetic resonance.

In the current study, Gd-DTPA was chosen because Gd-based contrast agents can significantly alter T1 relaxivity, which results in signal enhancement in T1 weighted images [15]. Electron microscopy has revealed that Gd can enter the maize root system and become distributed in the intercellular space [16]. Gd is a rare earth element (REE). REEs are micronutrients, which can enhance plant development and increase crop yields [17]. However, if the levels of REEs are too high, they may be toxic to plants. REEs have a hormesis effect on plants. Therefore, it is important to estimate the appropriate level of Gd that enables normal growth in rice. Also, it is important to study the changes in signal intensity in rice root MRI using the contrast agent Gd-DTPA.

In the present study, we used MRI to image rice roots treated with different levels of Gd-DTPA. To determine the impact of Gd-DTPA on MRI image quality and rice root biology, we measured signal intensities and spin-lattice relaxation times (T1) and examined the biological effects of Gd-DTPA on rice roots.

## Materials and Methods

### Plant Materials and Cultivation

The rice (*Oryza sativa* L.) cultivar used in this study was Wuyunjing 7 (*japonica*, introduced from Wujing Agriculture Research Institute in Jiangsu province, China). The materials included mutant-type (MT) and wild-type (WT) rice. The MT rice employed in this study has a spiral root system.

Rice seeds were sterilized in 1% NaClO for 20 min and washed with deionized water. The seeds were then germinated on wet filter paper in Petri dishes for 3 days at 30°C. Same-sized seedlings were chosen for hydroponic cultivation. The hydroponic nutrient solutions were replaced every 3 days to avoid a lack of nutrition and to ensure proper root growth. Plants were grown in a programmable illuminated incubator with a daily cycle of 12 hours light/12 hours darkness at a controlled temperature of 30°C day/26°C night.

### Gd-DTPA Treatments

After the rice was grown for two weeks, same-sized seedlings were chosen for analysis. The liquid adsorbed on the root surface was removed by washing the roots with deionized water several times. The plants were then subjected to treatments with different concentrations (0 mmol, 2 mmol, 5 mmol, 10 mmol and 15 mmol) of Gd-DTPA solution for different periods of time, i.e. three hours (3 h), six hours (6 h), nine hours (9 h) and twelve hours (12 h). Each treatment had three replicates.

### Assessment of the Short-term Effects of Gd-DTPA on Plant Growth

To estimate the appropriate level of Gd for normal plant growth, electrical conductivity and plant height were examined. Electrical conductivity (EC) was used to characterize the membrane permeability of the Gd-treated root samples [18]. Rice root systems immersed in Gd-DTPA solutions were washed with distilled water and dried to remove surface water. The dried roots were weighted and cut into segments and soaked in beakers containing 50 ml deionized water for 12 hours at room temperature after which the first EC was recorded. The beakers were then immersed in boiling water for 10 min and the second EC was recorded. The results were calculated and expressed in µS cm^−1 ^g^−1^.

To observe the dynamic changes in plant height in response to Gd-DTPA treatment, Gd-DTPA was added to hydroponic solution and plant heights were measured on the third, fifth and seventh day of culture. No Gd-DTPA was added to the hydroponic solution for the control.

### Plant Sample Preparation for MRI

The rice roots were immersed in different levels of Gd-DTPA for different periods of time, and Gd-DTPA adsorbed on the root surface was washed with deionized water several times. The treated root segments were introduced into five separate capillaries, which were bound together with Parafilm. The capillaries were then inserted into a 5 mm NMR tube (Figure S1), which was constructed to help analyze the different signal intensities and T1 at the same time and in the same cross section. The NMR tube was manually truncated to ensure that the root segments were located within the radio-frequency (RF) coils (Figure S1A).

### MRI Experiments

All MRI experiments were performed on a 14.1 Tesla (600 MHz) 8.9 cm wide bore, actively screened, vertical bore MR spectrometer (Bruker Biospoin GmbH, Germany). T1 weighted images were acquired using a two-dimensional modified driven equilibrium Fourier transform (MDEFT) pulse sequence. The following key sequence parameters were chosen: TR/TE/TI = 1,500/3.7/1,000 ms; nominal excitation flip angle = 12°; FOV = 6 mm×6 mm and matrix = 128×128 to give 47 µm×47 µm spatial resolution (pixel size); slice thickness = 0.5 mm (40 slices, gap = 0); NEX = 5. The scan time was approximately 40 minutes. A series of inversion-prepared fast spin echo images were acquired for longitudinal relaxation time (T1) measurement, which were identical in all aspects (TR 6,000 ms, TE 5 ms, BW 25 kHz, slice thickness 2 mm, matrix 96×96, NEX 1) except for 20 inversion times (TIs), which varied linearly from 10 to 2,500 ms.

ImageJ software was used to analyze and display the images. Briefly, for each root, regions of interest (ROIs) were manually drawn around the entire contrast-enhancing portion in five non-continuous sections at equal intervals of 1 mm, and the areas and signal intensities were measured. The mean signal intensities were normalized to the ROI areas and calculated by averaging over the five sections. For T1 measurements, signal intensity (SI) versus TI relationships were fit to the following exponential T1 decay model by nonlinear least squares regression: SI (TI) = A1*exp (−TI/T1)+SI (0)

### Gd Detection

Inductively coupled plasma optical emission spectroscopy (ICP-OES; Thermo Fisher Scientific, USA) confirmed the Gd uptake and concentrations in various root samples. The Gd-treated roots were washed and blotted dry. Then 0.1 g dried roots were weighted and digested in 4 mL HNO_3_+0.25 mL H_2_O_2_+0.5 mL HClO_4_ under low temperature conditions and dissolved in 5 mL 2% HNO_3_ according to Zhang et al. [19]. Finally, the volume of each sample was adjusted to 25 mL. A solution of 1,000 ppm Gd was used as the analytical standard. The detection limit of Gd was 1 ng.

### Long-term Biological Effects of Gd Treatment in Rice

A pot experiment was utilized to determine the long-term biological effects of Gd on rice growth. Seeds were germinated and same-sized seedlings were chosen for cultivation. The seedlings were cultivated in sandy soil (quartz sand and paddy soil with a proportion 2∶1 [V/V]) or paddy soil and treated with Gd-DTPA. Gd was added into the growth media to ensure the Gd content in media that was balanced according to the proportion between Gd-DTPA and media. Then the plant height, median root number, root biomass and tiller number were measured to determine the response of rice to Gd treatment. The plant height was recorded weekly during all growth periods. After the rice matured, the root systems were washed clear of soil and then a range of architectural traits were quantified. All of the samples were compared with control samples grown in the absence of Gd-DTPA.

### Statistical Analysis

The experiments examining the effects of Gd on rice growth and root imaging employed 3–5 samples, and each experiment had three replicates. Analysis of variance was carried out using Microsoft Origin software. Duncan’s multiple range test was performed to confirm the significance of the differences among the data for median root number, root biomass and tiller number (*P*<0.05) using SPSS statistical software.

## Results

### Examining Changes in EC to Determine Membrane Permeability

**Figure 1:**
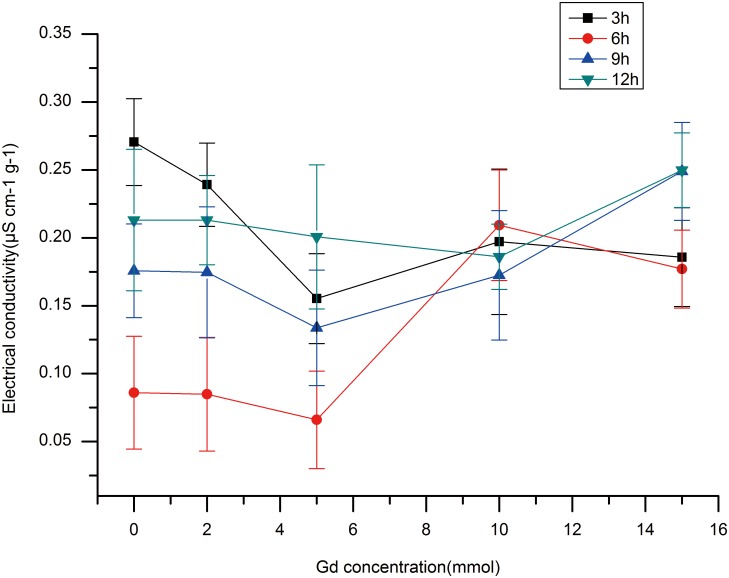
Differences in electrical conductivity (EC) in root samples of rice plants treated with different levels of Gd-DTPA for different periods of time.

EC is considered to represent membrane permeability. Lower EC levels indicate less damage to the membrane. The EC data from root samples treated with different concentrations of Gd-DTPA for different periods of time are shown in Figure 1. In all treatments, the EC of 5 mmol Gd-treated root samples was the lowest at 6 h, and at 3 h and 9 h, the EC value was lower in samples treated with 5 mmol Gd-DTPA than in the others. However, after 12 h Gd-DTPA treatment, there was little change in EC, and the 10 mmol Gd-treated root samples had the lowest EC values. Figure 1 showed that at all four time points, the EC first declined, then increased at higher Gd levels. Therefore, the lower concentrations of Gd-DTPA did not harm the membranes and to a certain extent had positive impacts on membrane permeability.

### Comparison of the Short-term Dynamic Changes in Plant Height

**Figure 2:**
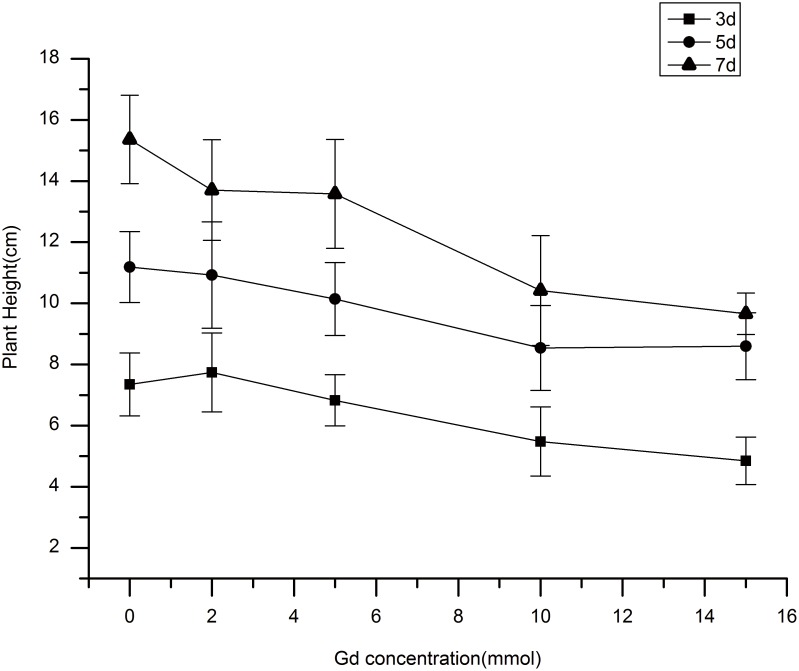
Comparison of plant heights after short-term treatment with different concentrations of Gd-DTPA solution.

Same-sized seedlings were chosen for hydroponic cultivation. Different concentrations of Gd-DTPA were added to the hydroponic solution, and the solution was replaced daily in order to ensure stable Gd concentrations. The dynamic changes in plant height are shown in Figure 2. Compared to the control concentrations of 2 and 5 mmol Gd-DTPA had little impact on plant height, but 10 and 15 mmol Gd-DTPA partially affected the normal growth of rice, leading to smaller increases in plant height compared to the other samples. Over time, the higher levels of Gd had more obvious effects on plant growth. These results indicate that 5 mmol Gd-DTPA is the optimal concentration for rice growth in the short-term.

### Evaluation of Gd-DTPA and Root Signals by MRI

**Figure 3:**
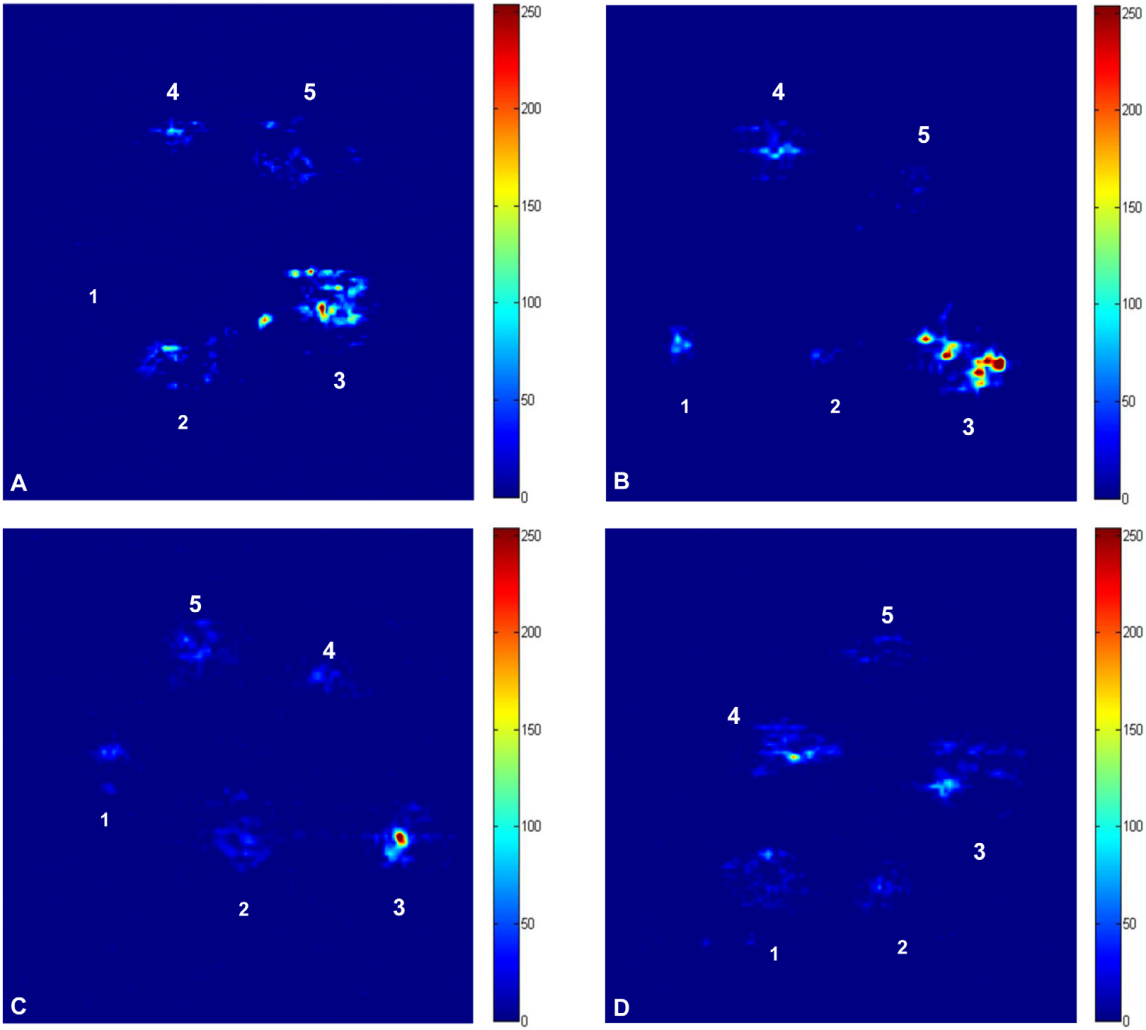
MRI images (transverse slices) of rice root samples immersed in different concentrations of Gd-DTPA for 3 h (A), 6 h (B), 9 h (C) and 12 h (D). In each image, numbers 1 to 5 represent 0-DTPA, respectively.

MRI images of rice root samples treated with different levels of Gd-DTPA were taken at four time points, including 3 h, 6 h, 9 h and 12 h (Figure 3).

After 3 h of treatment (Figure 3A), the brightness and colors of the images differed. It was easy to distinguish between the control and Gd-treated root samples, and 5 mmol Gd-treated root samples exhibited the maximum signals. However, it was difficult to distinguish between the other samples. As the processing time increased, the absorption of Gd and water increased. At 6 h (Figure 3B), the 5 mmol Gd-treated root samples were significantly brighter than the other samples, suggesting that the appropriate concentration of Gd-DTPA for rice root MRI is 5 mmol. The same phenomenon was observed at the 9 h time point (Figure 3C). However, there was no recognizable difference between the control root samples and the 2 and 10 mmol Gd-treated samples (Figure 3B, C). At 12 h (Figure 3D), the 5 and 10 mmol Gd-treated samples had better signals than the other samples, but it was difficult to differentiate the other root samples.

**Figure 4:**
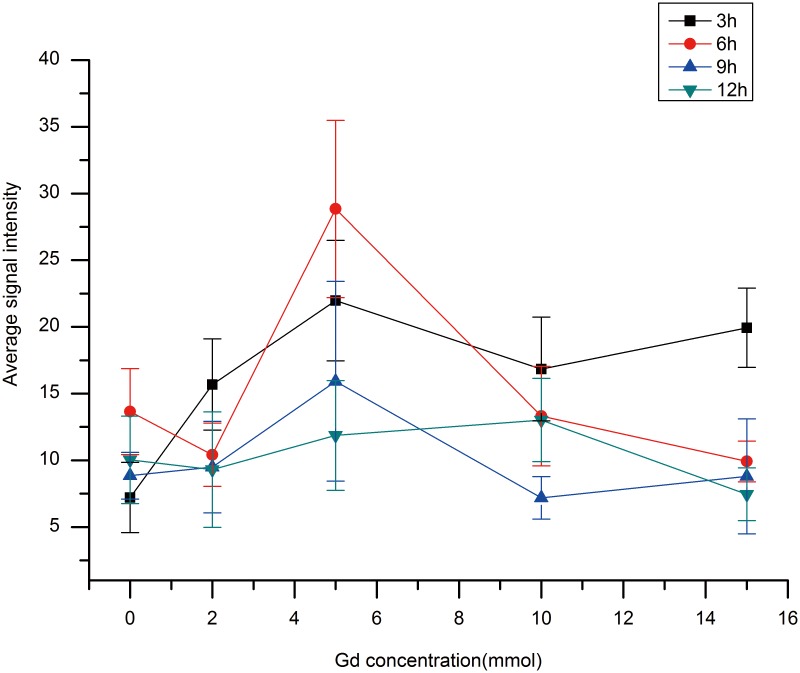
Average signal intensity (ASI) values of root samples that treated with different concentrations of Gd-DTPA at all four different time points.

We measured the average signal intensity (ASI) values of the root samples (Figure 4). Figure 4 showed that the ASI of the control sample was the lowest after 3 h of Gd-DTPA treatment. This result is in accordance with the MRI image (Figure 3A). Moreover, Figure 4 showed that 5 and 15 mmol Gd-DTPA had similar effects at 3 h, and the ASI values of these samples were similar. The MRI images of these samples were difficult to distinguish from each other. At 6 h, the ASI of 5 mmol Gd-treated samples was higher than that of the other samples (Figure 4), which was consistent with the MRI images (Figure 3B). Figure 4 showed that the trend of the differences in ASI at 9 h was similar to the signal differences observed at 3 h. At 12 h, there was no obvious difference in ASI between the samples, although the values appeared to be higher in the 10 mmol samples than in the 5 mmol samples (Figure 4).

**Figure 5:**
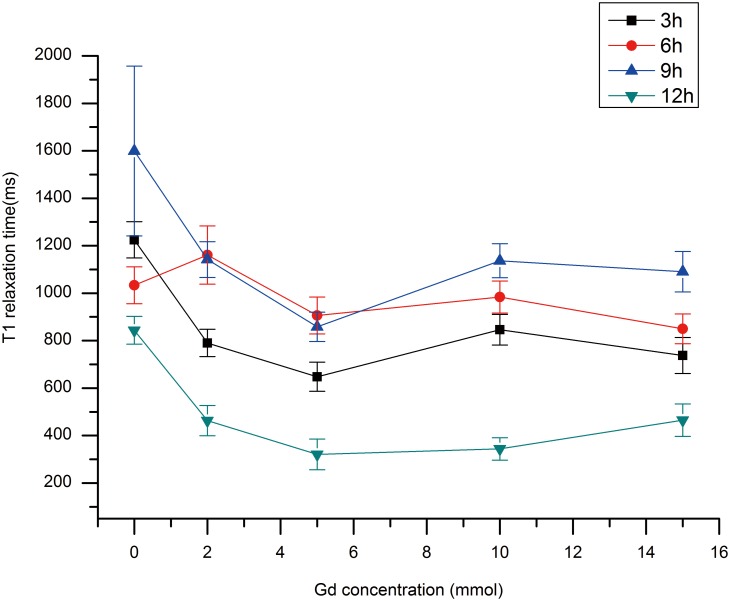
Spin-lattice relaxation times (T1) of root samples that treated with different concentrations of Gd-DTPA at different time points.

Compared to the T1 relaxation time of roots grown in the absence of Gd, i.e. 0 mmol, the T1 values of roots grown in different concentrations of Gd-DTPA decreased significantly, which suggests that Gd affects the relaxation rates of free water in the roots. The 5 mmol concentration yielded the lowest T1 values at all four time points, including 3 h, 6 h, 9 h and 12 h (Figure 5), suggesting that 5 mmol is the most appropriate concentration for root imaging. Compared to the changes in signal intensity (Figure 4), the opposite trend was observed for T1 (Figure 5). The results verified the accuracy of the MRI experiment and showed that 5 mmol Gd-DTPA is the best concentration to employ for MRI of rice roots.

### Concentration of Gd in Gd-treated Root Samples

**Figure 6:**
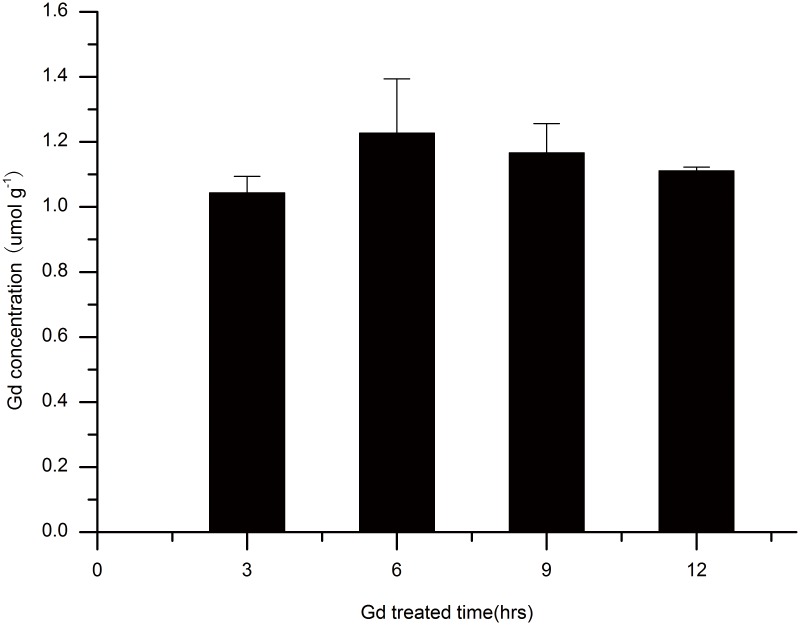
Concentrations of Gd in rice roots treated with 5-DTPA at different time points.

Previous experiments have shown that 5 mmol Gd-DTPA had a better effect on the signal intensity and T1 than the other concentrations. Physiological analysis of the effects of Gd-DTPA on rice roots demonstrated that 5 mmol Gd-DTPA had little impact on (or even enhanced) the growth of rice. However, at 6 h, Gd-DTPA had a stronger effect on EC and ASI. We then measured the Gd concentrations in rice roots exposed to 5 mmol Gd-DTPA (Figure 6).

For plants, treated with 5 mmol Gd-DTPA solution, the Gd concentrations in the roots obviously varied with treatment time. At 6 h, the Gd concentration reached its highest level, while the Gd concentration dropped a bit with increasing treatment time, suggesting that in roots, Gd accumulation tended to reach saturation at 6 h, or perhaps Gd is transported from the root to other parts of the plant. Moreover, this phenomenon may be due to the hormesis effect of Gd on roots.

### Structural Image of Rice Root Treated with 5 mmol Gd-DTPA for 6 h

**Figure 7:**
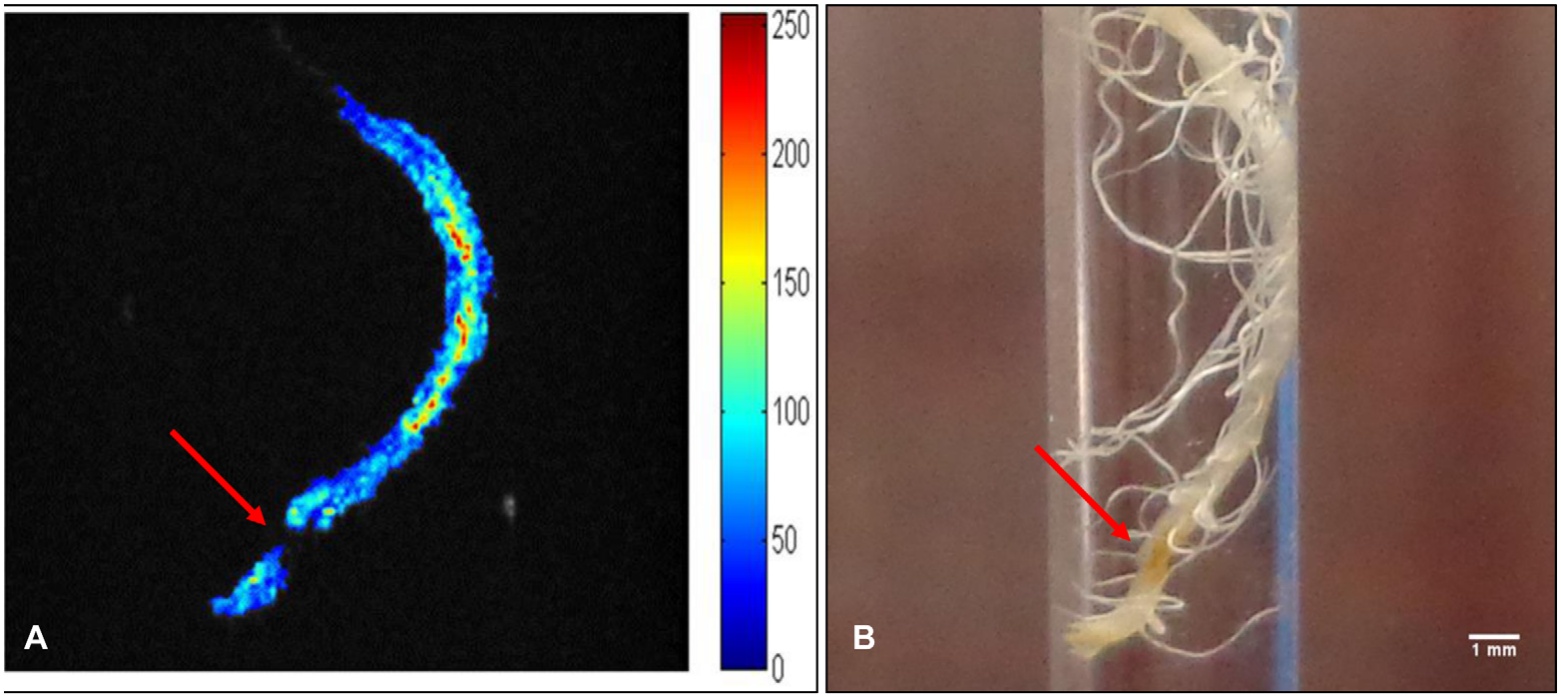
MRI image of rice root immersed in 5-DTPA for 6 h (A). Aging or dead tissues is marked with arrows. Image B shows a photograph image of the root sample.

From the above experiments, we determined that the appropriate Gd-DTPA treatment for structural imaging is 5 mmol for 6 h, a sample 3D image is shown in Figure 7. In Figure 7A, the taproot was cleared, and an obvious breakage had occurred. Compared to the photograph of the NMR tube (Figure 7B), little aging or dead tissue was evident in the MRI image. Since MRI technology is non-destructive, this image shows the potential applicability of this technique for detecting hidden root architecture.

### Long-term Biological Effects of Gd-DTPA on Rice

**Figure 8:**
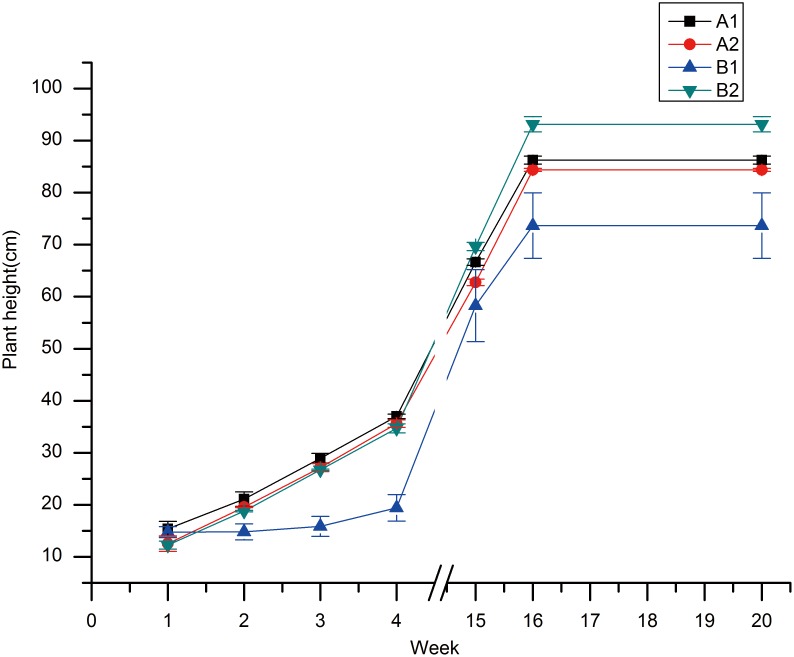
Treatment with 5-DTPA in different growth media. The plant height was altered at different growth periods. The growth media included sandy soil and paddy soil. A1 and B1 represent sandy soil and paddy soil treated with Gd-DTPA, and A2 and B2 represent untreated sandy soil and paddy soil, respectively.

The dynamic changes in plant height are shown in Figure 8. In this figure, 1 to 4 weeks represent the seedling stage of rice, 15 to 16 weeks represent the tillering stage and 16 to 20 weeks represent the heading and mature stages. Figure 8 showed that in sandy soil, the plant heights were slightly greater in the Gd-treated samples than in the control. However, the plant heights were clearly greater in standard paddy soil. Therefore, Gd partially affects the normal growth of rice, with less of an increase in plant height observed in paddy soil. However there was little distinction between the Gd-treated sample grown in sandy soil and the corresponding sample grown in paddy soil. After the rice matured, the root systems were washed clear of soil and a range of architectural traits were quantified (Table S1). The results showed that various aspects, especially the tiller number, were greater in Gd-treated samples than in the control. Therefore, treatment with 5 mmol Gd-DTPA had some enhancing effects on rice growth.

## Discussion

MRI is a non-destructive technology that can be used to visualize root architecture. To date, there are several reports on root imaging using MRI [11], [20], [21]. Compared to the rice root system, the systems examined in previous studies were larger and easier to observe. In the current study, the signal intensities in rice root MRI images were quite low. This study demonstrates the potential of Gd-DTPA for enhancing the signal intensity of MRI and its toxicological effects on rice. Zhong et al. [13] used the paramagnetic agents GdDTPA^2–^ and DyDTPA-BMA to enhance the images of maize root fragments, employing a Gd-DTPA^2–^ concentration of 10 mmol. According to this study, the concentration of Gd-DTPA was set around 10 mmol. In the current study, we found that the optimal concentration for observing rice roots by MRI is 5 mmol. Zhang et al studied the absorption of different concentrations of Gd in tomato plants and found that the Gd concentration in plant samples obviously increases in response to treatment with lower concentrations of Gd and increases slightly in response to higher concentrations [19]. These results are in accordance with our observation that the effect of Gd-DTPA on the growth of rice varied with the concentration of the treatment. We found that Gd uptake was restricted to samples treated with distinct levels of Gd-DTPA. To help explain this result, it is necessary to consider the factors that affect the ASI and T1 of root MRI. Gd-DTPA, one of the most popular MRI contrast agents, can drastically shorten T1 relaxation times in tissue and can enhance MRI signals in T1-weighted imaging. In this study, the increase in ASI and shortened T1 suggest that more Gd-DTPA has been deposited in the roots. Factors that affect the growth of rice should also be considered. First, the capacity of rice roots to adsorb Gd-DTPA did not increase with time, perhaps because the adsorption of roots reached saturation levels and Gd transport from roots to the other parts of the plant. Second, treatment with other concentrations of Gd solution affected the normal growth of rice, leading to less deposition of Gd-DTPA within a sample.

In addition to signal enhancement by Gd-DTPA, the T2 map demonstrated that different T2-values can be used to increase the signal-to-noise ratio (SNR) [22]. Moreover, different tissues have different relaxation times, making it easy to discriminate between the vacuole, cytoplasm and cell wall/extra-cellular space [23]–[25]. The combination of Gd-DTPA treatment and T2 mapping may help improve the quality of root images and enable different tissues to be distinguished.

The current study was primarily carried out under hydroponic conditions. However, rice should grow in the soil, and standard soil is not a good substrate for MRI studies: ions, impurities and holes in the soil may alter the magnetic environment, making the image quality in each slice different [26]. In addition, when Gd-DTPA is added to the soil, it is difficult to ensure that this compound will be present throughout the soil at proper levels, and precipitation of Gd solution in the soil may affect the spatial and temporal resolution of MRI. Perhaps an atomizing nozzle can be used to spray Gd-DTPA on the leaves of plants to avoid this problem. However, the transport mechanism of Gd from the aboveground parts of the plant to the roots remains under unclear. Paramagnetic elements in the soil such as Fe^2+^, Fe^3+^, Mn^2+^ and Cu^2+^ may further complicate MRI signal. All of these factors limit the applicability of Gd-DTPA and MRI techniques to soil research. It will be necessary to consider the properties of soil in future studies. It will be best to choose soil medium with fewer paramagnetic elements and homogeneous internal surroundings.

## Conclusions

This is the first report of imaging the root architecture in rice by non-destructive MRI using Gd-DTPA as a contrast agent to enhance signals. Since Gd is not required for rice growth and it has a hormesis effect on rice growth, some growth parameters were chosen as indicators. The ASI and T1 results and long-term biological effects of Gd treatment in rice suggest that 5 mmol Gd-DTPA is an appropriate concentration for root MRI. Further experiments are needed to broaden the choice of growth media and to elucidate the transport mechanism of Gd-DTPA in order to detect the root architecture of rice in soil by using MRI.

## Supporting Information

Figure S1 Plant samples preparation for MRI and schematic view of an NMR tube. Root segments treated with different concentrations of Gd were introduced into five separate capillaries, which were bound together, and inserted into a 5 mm NMR tube (A). Image B shows a schematic view of five separate capillaries in a 5 mm NMR tube.
(TIF)

Table S1 Architectural traits of rice under different processing conditions.
(DOC)
