# Correction: Effects of the Magnetic Resonance Imaging Contrast Agent Gd-DTPA on Plant Growth and Root Imaging in Rice

**DOI:** 10.1371/journal.pone.0120690

**Published:** 2015-04-02

**Authors:** The PLOS ONE Staff

The following information is missing from the Funding section: “the National Natural Science Foundation of China (No. U1232212; No. 31301297) (http://www.nsfc.gov.cn/).”
