# Supplementary figures and images for: Effects of the Magnetic Resonance Imaging Contrast Agent Gd-DTPA on Plant Growth and Root Imaging in Rice
Source: PLoS One. 2014 Jun 19;9(6):e100246. doi: 10.1371/journal.pone.0100246 (PMC4063760; doi:10.1371/journal.pone.0100246)

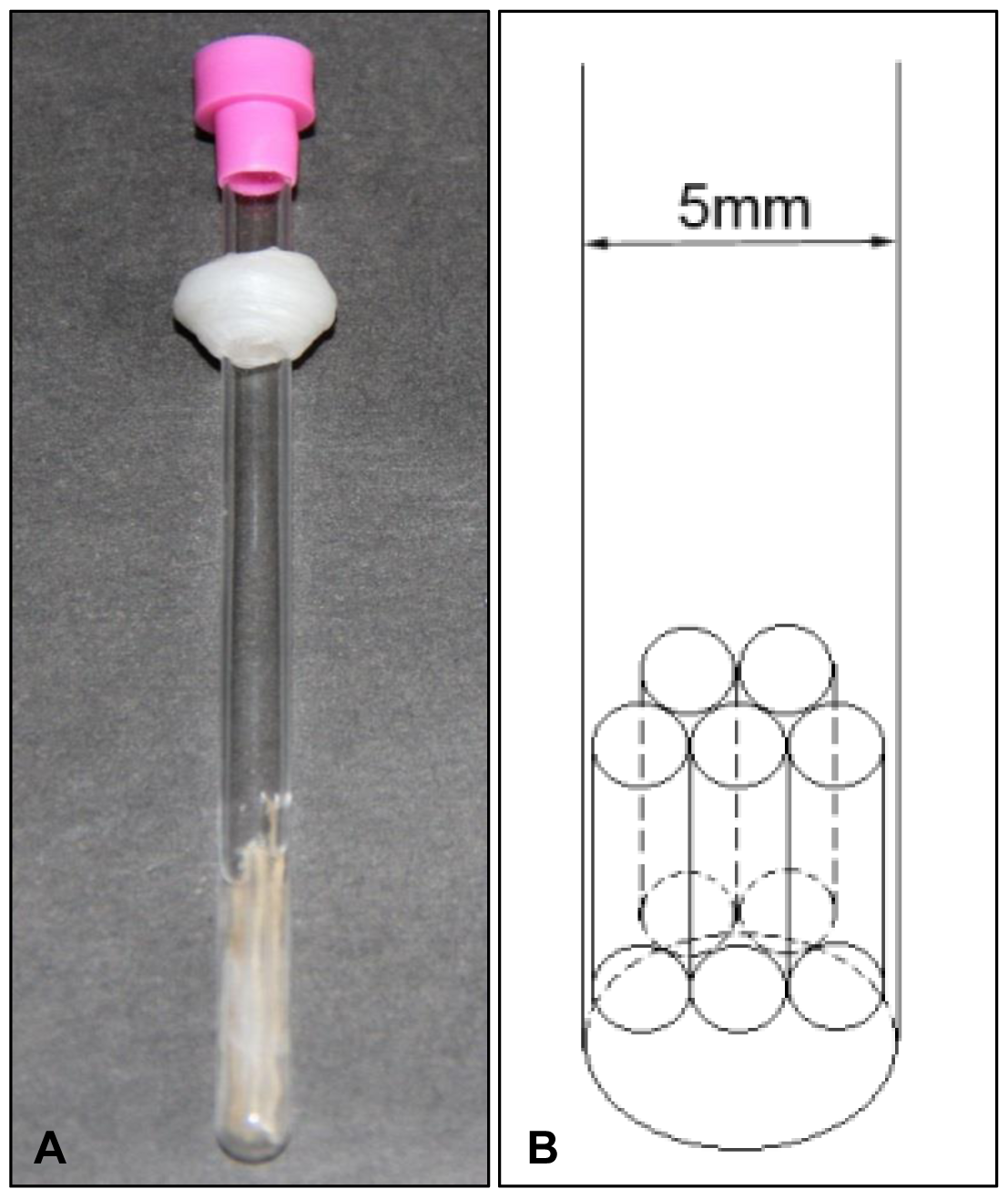

Supplement: Figure S1 — Plant samples preparation for MRI and schematic view of an NMR tube. Root segments treated with different concentrations of Gd were introduced into five separate capillaries, which were bound together, and inserted into a 5 mm NMR tube (A). Image B shows a schematic view of five separate capillaries in a 5 mm NMR tube. (TIF) [file pone.0100246.s001.tif]
