# Supplementary material for: Effects of the Magnetic Resonance Imaging Contrast Agent Gd-DTPA on Plant Growth and Root Imaging in Rice
Source: PLoS One. 2014 Jun 19;9(6):e100246. doi: 10.1371/journal.pone.0100246 (PMC4063760; doi:10.1371/journal.pone.0100246)
Supplement: Table S1 — Architectural traits of rice under different processing conditions. (DOC) [file pone.0100246.s002.doc]

**Table S1 A**rchitectural traits of rice that under different processing conditions.

| Trait | Sandy soil (Gd) | Sandy soil (con) | Paddy soil (Gd) | Paddy soil (con) |
| --- | --- | --- | --- | --- |
| Median root number | 288±44.23 a | 192.7±25.50 b, c | 166±13 c | 243±9.64 a, b |
| Root biomass (g) | 3.91±0.57 | 3.10±0.58 | 3.06±0.50 | 3.4±0.39 |
| Tiller number | 13.33±0.58 a | 5.67±0.58 b | 10.67±2.31 c | 7.33±1.15 b |

The means ±SE are shown. Different letters indicate a significant difference according to Duncan’s test (P < 0.05; n = 3).
